# A Manual of Elementary Chemistry, Theoretical and Practical

**Published:** 1845-01

**Authors:** 


### A MANUAL OF ELEMENTARY CHEMISTRY, THEORETICAL AND PRACTICAL. By *George Fownes*, PH. D. Chemical Lecturer in the Middlesex Hospital Medical School, and to the Pharmaceutical Society of Great Britain. Pp. 566. London, 1844.

NUMEROUS and useful as are the works extant on the science of chemistry, we are nevertheless prepared to admit that the author of this publication has made a valuable addition to them, by offering the student and those in general who desire to obtain information, an accurate compendium of the state of chemical science; which is, moreover, well illustrated by appropriate and neatly-executed wood-engravings.

At the commencement of his Preface the author has so clearly and appositely expressed the view which he entertained in compiling his work, that we cannot do better than give his own words.

> "The design of the present volume is to offer to the student commencing the subject of Chemistry, in a compact and inexpensive, but, it is hoped, not unintelligible form, an outline of the general principles of that science, and a history of the more important among the very numerous bodies which Chemical investigations have made known to us. The work has no pretensions to be considered a complete treatise on the subject, but is intended to serve as an introduction to the larger and more comprehensive works in our own language and in those of the Continent."

Dr. Fownes has divided his work into four Parts—the first treats of Physics, including *Density and Specific Gravity; The Physical Constitution of the Atmosphere, and of Gases in general*, and *Heat, Light, Electricity*, and *Magnetism;* the second Part treats of *Non-metallic Elements;* the third, of *Metals;* and the fourth, of *Organic Chemistry;* this last appears to be the favourite portion of the author's subject, for of 540 pages of which his work consists, 200 are occupied by it alone; we indeed are of opinion that, considering the work as intended to be elementary, the author would have done better to have dedicated some portion of the space which he has bestowed upon organic chemistry to a more full consideration of chemical affinity, and the circumstances under which it is excited and exerted, and those by which it is accelerated, impeded, or prevented.

Unless we mistake, no mention is made of cohesion in general, or of the effect which it has of controuling the chemical action of bodies upon each other; the chemical agency of light and electricity, might also, we think, have been advantageously considered more at length.

When electricity, the last of the imponderable bodies, has been treated of, (and of this portion of the work we have no complaint to offer), the author naturally proceeds to the enumeration of the elementary ponderable bodies, which he subdivides into the three classes of *non-metallic, intermediate*, and *metals*. It appears to us that there was no necessity for, nor any advantage gained by introducing the second sub-division; it includes phosphorus, arsenic, and tellurium; why the first should not be considered as perfectly non-metallic as sulphur, or why, if antimony be a metal, arsenic is not, we cannot discover. With respect to tellurium, indeed, we observe that Dr. Fownes has adopted an antiquated term in treating of it at p. 310—he calls it "this metal or semi-metal." We do not find that the author has stated any reasons for his arrangement, though we are far from asserting that he could not have assigned satisfactory ones.

The substance first treated of in detail is Oxygen, and as we do not know that we can give a more fair specimen of Dr. Fownes's manner of treating of the subjects which he has to describe, than by quoting this part of his work, we shall give a considerable portion of his statements with respect to oxygen. We shall take the liberty of appending to it a few remarks, which we trust will be received with the same friendly spirit as that by which they are dictated.

"Oxygen was discovered in the year 1774, by Scheele, in Sweden, and Dr. Priestley, in England, independently of each other, and described under the terms *empyreal air* and *vital air*. The name oxygen* was given to it by Lavoisier some time afterwards. Oxygen exists in a free and uncombined state in the atmosphere, mingled with another gaseous body, nitrogen; no direct means exist, however, for separating it from the latter, and accordingly, it is always obtained for purposes of experiment by decomposing certain of its compounds, which are very numerous.

"The red oxide of mercury, or *red precipitate* of the old writers, may be employed with this view. In this substance the attraction which holds together the mercury and the oxygen is so feeble that simple exposure to heat suffices to bring about decomposition. The red precipitate is placed in a short tube of hard glass, to which is fitted a perforated cork, furnished with a piece of narrow glass tube, bent as in the figure. The heat of a spirit-lamp being applied to the substance, decomposition speedily commences, globules of metallic mercury collect in the cool part of the wide tube, which answers the purpose of a retort, while gas issues in considerable quantity from the apparatus. This gas is collected and examined by the aid of the pneumatic trough, which consists of a vessel of water provided with a shelf, upon which stand the jars or bottles destined to receive the gas, filled with water and inverted. By keeping the level of the liquid above the mouth of the jar, the water is retained in the latter by the pressure of the atmosphere, and entrance of air prevented. When brought over the extremity of the gas-delivering tub, the bubbles of gas arising through the water collect in the upper part of the jar and displace the liquid. As soon as one jar is filled, it may be removed, still keeping its mouth below the water-level, and another substituted.

"The experiment described is more instructive as an excellent case of the resolution by simple means of a compound body into its constituents, than valuable as a source of oxygen gas. A better and more economical method is to expose to heat in a retort, or flask furnished with a bent tube, a portion of the salt called chlorate of potash. A common Florence flask serves perfectly well, the heat of a spirit lamp being sufficient. The salt melts and decomposes with ebullition, yielding a very large quantity of pure oxygen gas, which may be collected in the way above described. The white saline residue in the flask is chloride of potassium. This plan, which is very easy of execution, is always adopted when very pure gas is required for analytical purposes.

"A third method, very good when perfect purity is not demanded, is to heat to redness in an iron retort or gun-barrel, the black oxide of manganese of commerce, which under these circumstances suffers decomposition, although not to the extent manifest in the red precipitate.

---

* "From ὀξὺς, acid, and γεννάω, I give rise to."

"If a little of the black oxide of manganese be finely powdered and mixed with chlorate of potash, and this mixture heated in a flask or retort by a lamp, oxygen will be disengaged with the utmost facility, and at a far lower temperature than when the chlorate alone is used. All the oxygen comes from the chlorate, the manganese remaining quite unaltered. The materials should be well dried in a capsule before their introduction into the flask. This experiment affords an instance of an effect by no means rare, in which a body seems to act by its mere presence, without taking any obvious part in the change brought about." 93.

In his brief account of the discovery of oxygen Dr. Fownes, quite unintentionally, we doubt not, has done some injustice to Dr. Priestley. It would seem, from the mode of statement, and Scheele's name being placed before that of Priestley, that both of them had made the discovery in the same year, and Scheele the earlier of the two. That Dr. Priestley discovered oxygen in 1774 is universally admitted, for he exhibited its properties to Lavoisier in that year, and in 1775 appeared his work containing an account of the mode in which he procured it. Now Scheele's work on Air and Fire, containing the account of his discovery of the gas, was not published till 1777. Again, Dr. Priestley described it under the name of *diphlogisticated air*, while it was Scheele who called it *empyreal air*, and Condorcet who named it *vital* air.

The author has noticed the bad conducting power of water with regard to electricity, but he does not mention its bad conduction of heat under the head of water, though allusion is made to it when treating of conduction; this curious subject might, we think, have been advantageously dwelt upon a little more at length.

There is an observation at p. 108, which we suspect conveys more than the author could have meant; he observes, that "the ocean is the great recipient of the saline matter carried down by the rivers, which drain the land; hence the vast accumulation of salts." This language conveys to us the idea that the author supposes the saline contents of the sea to have originated from the saline matter carried down by the rivers, an opinion, which we presume he will hardly maintain, though it undoubtedly contributes to increase the quantity.

With respect to nitric acid, we may observe that Dr. Fownes has stated the composition of nitric acid of 1·5 density, incorrectly at 54·06 real acid and 9 water. Dr. Ure's experiments show very nearly that it consists of one equivalent of nitric acid 54 and one and a half of water 13·5; and we may here remark that the author, notwithstanding the evidence which has recently been adduced in favour of nitrogen having 14 for its atomic weight, represents it by 14·06. A few mistakes of the nature which we have just pointed out, could hardly fail to occur in a work containing so many facts as one on chemistry necessarily does; we doubt not, however, that before a second edition is called for, the author will have carefully revised what he has written, and corrected such slight mistakes as we have noticed.

To that part of the subject which relates to organic chemistry, Dr. Fownes has evidently paid much and minute attention. We find it difficult to select any particular portion for approval, but we may remark, that the subject of ether has been very ably treated of, and that the facts relating to ethereal compounds are compressed with clearness and accuracy into a very small compass.

After what we have stated of this work, our readers will not be surprised that it has our hearty commendation, and that, in our opinion, it is calculated, and at a trifling expense, to spread the doctrines of the intricate science which it so clearly explains.

